# A combinatorial approach to determine the context-dependent role in transcriptional and posttranscriptional regulation in *Arabidopsis thaliana*

**DOI:** 10.1186/1752-0509-3-43

**Published:** 2009-04-28

**Authors:** Le Lu, Jinming Li

**Affiliations:** 1 Division of Structural and Computational Biology, School of Biological Sciences, Nanyang Technological University, 60 Nanyang Drive, Singapore 637551, Singapore; 2 Bioinformatics Division, TNLIST and Department of Automation, Tsinghua University, Beijing 100084, PR China

## Abstract

**Background:**

While progresses have been made in mapping transcriptional regulatory networks, posttranscriptional regulatory roles just begin to be uncovered, which has arrested much attention due to the discovery of miRNAs. Here we demonstrated a combinatorial approach to incorporate transcriptional and posttranscriptional regulatory sequences with gene expression profiles to determine their probabilistic dependencies.

**Results:**

We applied the proposed method to microarray time course gene expression profiles and could correctly predict expression patterns for more than 50% of 1,132 genes, based on the sequence motifs adopted in the network models, which was statistically significant. Our study suggested that the contribution of miRNA regulation towards gene expression in plants may be more restricted than that of transcription factors; however, miRNAs might confer additional layers of robustness on gene regulation networks. The programs written in C++ and PERL implementing methods in this work are available for download from our supplemental data web page.

**Conclusion:**

In this study we demonstrated a combinatorial approach to incorporate miRNA target motifs (miRNA-mediated posttranscriptional regulatory sites) and TFBSs (transcription factor binding sites) with gene expression profiles to reconstruct the regulatory networks. The proposed approach may facilitate the incorporation of diverse sources with limited prior knowledge.

## Background

Transcription factors (TFs) regulate gene expression by binding selectively to DNA sequences in promoters, and genes regulated by the same TFs have been assumed to share the common binding sites in their promoter regions and exhibit similar expression patterns [1]. Numerous experimental and computational studies [2] have been done on locating transcriptional regulator DNA binding sequences and understanding their working mechanisms. These binding motifs can be used as building blocks of gene regulatory networks and several approaches were developed to identify how a set of *cis*-regulatory elements in a gene's promoter region governed its behavior and explained the observed expression profiles [3-5]. Using different approaches, Segal et al. [3] and Beer and Tavazoie [4] both showed that a substantial fraction of yeast gene expression profiles could be explained in terms of the combination of *cis*-regulatory elements. However, a limitation of such approaches is that many genes are posttranscriptionally regulated [3]. The progresses have been made in mapping transcriptional regulatory networks in recent years, whereas posttranscriptional regulatory roles just begin to be uncovered [6,7]. Posttranscriptional regulation through RNA-RNA interaction has arrested much attention due to the discovery of microRNAs (miRNAs).

miRNAs regulate gene expression by inducing mRNA cleavage or translational repression of their targets [8]. Plant miRNAs are usually perfectly complementary to their targets and cause the cleavage of their targets by RNA-induced silencing complex (RISC), whereas in animals targets with weaker complementarities appear to have decreased translational efficacy [9]. However, the role of miRNA in regulatory networks needs to be further explored [7]. To address this need, we introduced a combinatorial approach to determine the transcriptional and posttranscriptional regulatory elements based on gene expression profiles.

Various plant growth and development processes are critically influenced by light [10-12]. Wild type *Arabidopsis *seedling development follows two patterns, etiolation in darkness and photomorphogenesis in the light [13]. *COP/DET/FUS (CONSTITUTIVE PHOTOMORPHOGENIC/DE-ETIOLATED/FUSCA) *is a class of genes which were identified as downstream signalling components of all photoreceptors [14-16]. Mutation in *COP/DET/FUS *causes constitutive photomorphogenic development even in the dark [14,17]. One important light-signalling component involved in plant light responses is COP1 [14], which regulates not only photomorphogenesis but also other developmental processes. The constitutive photomorphogenic phenotype of *cop1 *mutation indicates that COP1 acts as a negative regulator of photomorphogenesis [13,18].

**Figure 1:**
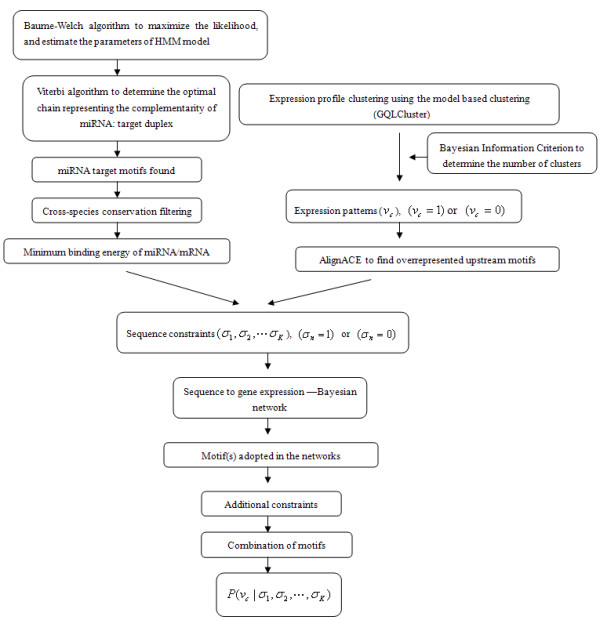
**Flowchart of the combinatorial approach to determine the transcriptional and posttranscriptional regulatory motifs based on gene expression profiles**. Firstly, we conducted a genome-wide screening to detect potential miRNA target motifs in *Arabidopsis *based on an inhomogeneous HMM and cross-species conservation and minimum binding energy of miRNA/mRNA duplex were used as additional filters to reduce the rate of false positives. Secondly, genes in the *cop1 *mutant time course microarray dataset were clustered into 12 expression patterns and overrepresented sequence elements in the upstream of the genes belonged to the same cluster were detected using AlignACE. Thirdly, Bayesian network strategy was applied to selecting these motifs in both upstream sequences and transcripts that were most related to the gene expression patterns. Lastly, we measured the degree to which gene expression could be determined merely by these adopted regulatory motifs.

We applied this approach to a *CONSTITUTIVE PHOTOMORPHOGENIC1 *(*COP1*) mutant time course microarray dataset to detect sequence elements that selectively bind to TFs and miRNAs in the process. Inspired by Beer and Tavazoie [4], we used Bayesian network – a probabilistic model to integrate gene expression profiles, transcription factor binding sites (TFBSs) as well as miRNA target motifs to deduce the combination of sequence elements that modulate gene expression, and we tried to explain the observed gene expression profiles in terms of the adopted motifs. Firstly, we conducted a genome-wide screening to detect potential miRNA target motifs in *Arabidopsis *based on an inhomogeneous Hidden Markov model (HMM), and cross-species conservation as well as minimum binding energy of miRNA/mRNA duplex were used as additional filters to reduce the rate of false positives. Secondly, genes in the *cop1 *mutant time course microarray dataset were clustered into 12 expression patterns and overrepresented sequence elements in the upstream of the genes belonged to the same cluster were detected using AlignACE [19]. Thirdly, Bayesian network strategy was applied to selecting these motifs in both upstream sequences and transcript sequences that were most related to the gene expression patterns. Lastly, we measured the degree to which gene expression could be determined merely by these adopted regulatory motifs. Figure 1 illustrated the flow diagram of the approach.

## Results

### miRNA target motifs in *Arabidopsis*

Various algorithms developed to predict plant miRNA targets are on the same basis that miRNAs and their targets are perfectly complementary, and most of the algorithms predict miRNA targets through detecting transcripts that have less than or equal to 4 mismatches to miRNAs [20]. However, there are natural targets with 5 mismatches [21], which are not able to be found by these algorithms. Moreover, we believe that sequences with the same number of mismatches to a miRNA might not have the same probability to be cleaved by the miRNA owing to the mechanism of RISC. In several cases, particular miRNA-target mismatches are conserved through the evolutionary distance that separates *Arabidopsis *and rice [22], suggesting that certain mismatches might be under positive selective pressure rather than merely being tolerated. Furthermore, properly placed mismatches might improve the enzyme turnover rate [22].

We chose HMM because of its capability of capturing the position specific information about particular matches/mismatches. In spite of the variable miRNA sequences, the complementarities between miRNA-target duplex might follow some rules according to the RISC mechanisms, and we believed that the HMM could be used to find these hidden rules by learning from a training set of potential miRNA targets of only 19 mature miRNAs contained in miRBase 3.0, a three years old release, and in this way we also assessed the ability of our method to extrapolate from a limited prior knowledge [23]. To obtain the training set, we set the maximum number of mismatches tolerated at 4, and the direct search detected 223 genes whose mRNAs had the complementary sites with at least one of the 19 miRNAs. The 223 miRNA-target candidates were used as training data. The Baum-Welch algorithm estimated the transition and emission probabilities and the optimal state chains of each of the miRNA-mRNA pair were computed using Viterbi algorithm, which represented possible miRNA-target duplexes that could be recognized by RISC and cleaved by its Argonaute component.

Totally 103 non-redundant optimal state chains were produced by using Viterbi algorithm, which were much less than the number of possible chains that randomly allowed up to 4 mismatches for a 20 mer mRNA. After scanning the genome, we found about 150,299 potential miRNA target motifs for all the 212 miRNAs in the miRBase newest release (Release 12.0). This result covered almost all the experimentally validated miRNA targets (90/91) in *Arabidopsis *[20,21,24]. To reduce the false positive rate of our HMM predictions we used the cross-species conservation and minimum binding energy of miRNA/mRNA duplex to do two rounds of filtering. There are 122, 844 HMM predictions passed through the first round of selection, and among them 30,451 passed through the second round of selection. Almost all of the 91 experimentally validated miRNA targets (90/91) passed through the first round of selection, and among them 75 passed through the second round of selection. The majority of the 91 experimentally validated miRNA targets (58/91) were the targets for those miRNAs that were not included in the training set.

We did simulation study by random shuffling of miRNA sequences to test whether our method could distinguish a miRNA from its shuffled version during the detecting process. Two kinds of randomly shuffled sequences were generated, i.e. *monoshuffled *and *dishuffled *sequences. The *monoshuffling *method generated a truly permuted random sequence while the *dishuffling *method further made the count of each dinucleotide the same as that of miRNAs. Fifty cohorts of randomly shuffled sequences were generated. The noise to signal ratio (the average number of predicted targets in 50 cohorts of randomly shuffled sequences *versus *the number of targets detected for authentic miRNAs) was 0.49 (*monoshuffling*) and 0.50 (*dishuffling*), respectively. The detailed simulation results are available in our supplemental web page .

### Discovery of transcriptional and posttranscriptional regulatory motifs in cop1 mutant time course microarray data

In the *cop1 *mutant time course experiment, there were in total 10 time points, i.e. 0^th ^hour, 12^th ^hour, 24^th ^hour, 36^th ^hour, 48^th ^hour, 60^th ^hour, 72^nd ^hour, 4^th ^day, 5^th ^day and 6^th ^day. The log expression ratio reflected the difference between the expression level of *cop1 *mutant and that of wildtype for each gene.

**Figure 2:**
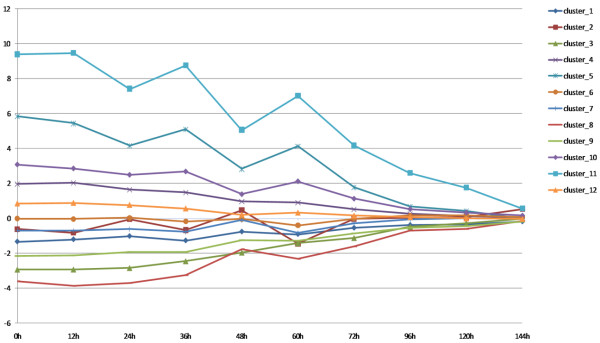
**Maximal log likelihood value obtained by BIC showed that the optimal number of clusters was 12, so we divided the 5,689 genes into 12 clusters using GQLCluster**. Each cluster contained 755, 157, 400, 509, 275, 638, 725, 374, 658, 422, 186 and 590 genes, respectively. The mean expression profiles for each of the 12 clusters were calculated and plotted.

Maximal log likelihood value obtained by BIC showed that the optimal number of clusters was 12, so we divided the 5,689 genes into 12 clusters using GQLCluster [25]. Each cluster contained 755, 157, 400, 509, 275, 638, 725, 374, 658, 422, 186 and 590 genes, respectively. The mean expression profiles were calculated for each cluster (Figure 2), and the 12 gene clusters and their mean expression profiles are available in our supplemental data web page . Sequences that were 3000 bp upstream of transcription start sites (TSSs) were retrieved for each gene and TFBSs were detected using AlignACE for the genes belonged to the same cluster. The computer program ScanACE  with default parameters was used to identify the TFBSs in the upstream region of each gene. The predicted TFBSs for each cluster are available in our supplemental data web page. We also added 15 known hexamer motifs described in Gao et al. [26] to the TFBS dataset.

The TFBSs and miRNA target motifs were fed to the Bayesian network model and the models weighted sequence motifs according to their contribution to the expression profiles. There had been no evidence that the TF binding to a gene's upstream region could also posttranscriptionally affect its cleavage by miRNA and vice versa, therefore the TFBSs and miRNA target motifs were treated independently in the network construction. No interaction is allowed between two motifs of different kinds. For TFBSs, their distances to TSSs, their orientations, copy numbers and the interaction between any two adopted TFBSs are all taken into account. Our microarray time course experiment was not specially designed to test miRNA targets expression, so we gave upstream motifs the priority in the network construction. Therefore, a network might only have upstream motif nodes without any miRNA target nodes, but could not only have miRNA target nodes instead. About 80% of the genes (4,557) were used to train the Bayesian network model and the rest 20% genes (1,132) were used to estimate the proportion of the genes whose expression patterns could be correctly predicted by merely the adopted transcriptional and posttranscriptional regulatory motifs in the networks.

The average number of nodes was 7 for the 12 networks, and in average 3 were upstream motif nodes and 4 were miRNA target nodes (listed in supplemental Tables S1 and S2). The most frequent constraints added to each TFBS node was its distance to TSS. Two known upstream motif nodes had been added, respectively, to two networks, namely MYB1At to network 8 and I-box to network 12. Totally 48 miRNA target nodes were adopted by the 12 networks.

### Predicting gene expression patterns

We used the upstream motif nodes and the miRNA target nodes adopted in the Bayesian network model to predict gene expression patterns. Each of the 1,132 genes was assigned to the respective network with the highest probability *p*(*v*_*c *_= 1|*D*, *S*_*c*_). Some expression patterns were quite similar; hence we calculated the correlation coefficient of the mean expression pattern between any two of the 12 clusters. If two expression patterns have a correlation coefficient greater than 0.9, they are regarded as overlapped expression patterns. We regarded overlapped expression patterns as in a single cluster, in this way we classified the 12 expression patterns into 4 qualitative distinguished super-clusters (Table 1). A gene assigned to the correct super-cluster would be regarded as correctly predicted [4].

**Table 1:** Gene expression patterns (clusters) in each of the four super-clusters

Super-cluster 1	Cluster 1, 3, 8, 9
Super-cluster 2	Cluster 3, 4, 9, 10, 11
Super-cluster 3	Cluster 5
Super-cluster 4	Cluster 6

More than 50% genes (569/1132) were correctly assigned. We did simulation study by randomly assigning the 1,132 genes to the 4 super-clusters for 100,000 times. The number of correctly assigned genes was 329 in average, and the P-value of correctly assigning 569 genes was less than 1e-05. Moreover, 552 out of the 569 genes could still be correctly assigned without miRNA nodes and the introducing of miRNA nodes could further correctly assigned 17 genes. We retrieved the functional annotation of these 17 genes and found that two genes (*At5g63460 *and *At5g67300*) have the annotation term "DNA or RNA binding" in the GO [27]. Furthermore, we made a 5-fold cross validation test and the average number of correctly assigned genes was 530.

## Discussion

### Transcriptional and posttranscriptional regulatory networks

We applied our method to shorter promoter regions; say 1000 upstream to 500 downstream of each TSS. And the accuracy of the expression pattern prediction with (-1000, 500) region is lower than that of (-3000, 0) region. Only 486 genes could be correctly assigned to its respective expression pattern; and without the integration of miRNA nodes, 474 genes could be correctly assigned merely based on TFBSs nodes.

Most genomic studies of gene expression regulation focus on transcriptional rather than on posttranscriptional regulation. Based on a model in which upstream motifs contribute additively to the log-expression level of a gene, Bussemaker presented a computational method [28] for discovering *cis*-regulatory elements that circumvented the need to cluster genes based on their profiles. Beer and Tavazoie [4] correctly predicted 70% of the gene expression patterns by use of Bayesian network only based on upstream motifs. Li et al. developed a promoter classification method using a Relevance Vector Machine (RVM) and Bayesian statistical principles to identify discriminatory features in the promoter sequences of genes that could classify transcriptional responses and they correctly predicted 70% genes as being up- or down-regulated [29], based on a small set of discriminative promoter motifs.

In the meanwhile, Foat et al. identified functional 3' UTR motifs (including miRNA target sites) that best correlated with the observed changes in mRNA levels [30,31]. Sood et al. used computational methods to explore the effects of endogenous miRNA expression on endogenous steady-state mRNA levels [32]. In their model, changes in mRNA levels of a given gene (measured by the microarray experiment) are written as a sum over contributions from all sequence motifs in the 3' UTR of that gene, which could explain changes in mRNA levels for 50% genes. In order to understand the importance of sRNAs in gene regulation, Levine et al. [6] studied examples from two distinct classes of bacterial sRNAs based on a quantitative approach combining experiments and theory. Their results suggested that sRNA provides a distinct mode of gene regulation from that of protein-mediated one.

Although Beer and Tavazoie as well as Rajewsky [4,31] all suggested the integration of posttranscriptional and transcriptional motifs in the future studies of gene regulatory networks, respectively,[4,30] none of the aforementioned groups had correlated both transcriptional and posttranscriptional regulatory elements together with the gene expression data. Recently Hobert [7] briefly reviewed the principles of TF and miRNA working mechanisms and how they control gene expression.

### Plant miRNA target prediction

In the Rhoades et al.'s study [33], random permutation was used to evaluate the performance of the proposed method of plant miRNA target prediction. Annotated *Arabidopsis *mRNAs were searched for targets for 16 *Arabidopsis *miRNAs. Identical searches with 10 cohorts of 16 randomized miRNAs were also performed. When constrained to 0–4 mismatches, 157 targets were predicted for 16 miRNAs, whereas in average 55.4 targets were predicted for the cohorts of random sequences, which gave a noise to signal ratio of 0.35 (55.4/157). When the number of mismatches was exactly 4 in their prediction method, the ratio became 0.53 (51/96). In our simulation study using two different shuffling methods (see Figure S1 in our supplemental web page), the noise to signal ratio (the average number of predicted targets in 50 cohorts of randomly shuffled sequences *versus *the number of targets detected for authentic miRNAs) were 0.49 (*monoshuffling*) and 0.50 (*dishuffling*), respectively.

If the number of mismatches allowed in Rhoades et al.'s method was 0 to 4, our method may generate more false positives (0.50 or 0.49 *versus *0.35), which might be due to the fact that our HMM method allows for more mismatches. However, when the number of mismatches was fixed at 4 in Rhoades et al.'s method, the noise to signal ratio increased to 0.53. Our proposed HMM method of plant miRNA target prediction allows for more than four mismatches in the target sequences, however, we proposed this method here as an alternative instead of a replacement of the published method, since the HMM method may increase the number of false positive predictions due to the allowance of more than 4 mismatches.

### Contribution of miRNAs in gene regulation networks

In our study, 3% of the 569 genes could only be correctly assigned after introducing miRNA nodes, which might suggest that the consequence of miRNA-mediated posttranscriptional regulation was marginal in our time course expression profiles though miRNA is considered as one of the most important posttranscriptional gene regulators. This might result from a possible bias in the predictive power of TFBS since the motif finding was done for each fixed cluster. In view of this, we did a reference test using only the aforementioned 15 known hexamer motifs [26] and miRNA target motifs. Using the 15 known hexamer motifs, we could only correctly assign 296 genes, which was even less than that from random assignment (P-value < 0.98) and this suggested that the observed expression profiles could not be explained solely by the combination of the 15 known motifs. After adding miRNA target nodes, we could correctly assign 509 genes (P-value < 1e-05). The result suggested that miRNAs might confer additional layers of robustness on gene regulation networks. Exploration of miRNA regulatory mechanism together with known transcriptional regulatory interactions and other functional genomics data might help to further elucidate the function of miRNAs at a system-wide level [7,31].

The 213 genes, which could only be correctly assigned once miRNA nodes were adopted, might have functions related to miRNA regulation mechanism. We retrieved the functional annotation of these 213 genes and found that three of them, namely *At5g12840*, *At5g60120 *and *At5g43780 *are experimentally validated miRNA targets. Furthermore, we grouped these genes based on their GO annotations (Table 2). It is not surprising to find that both functional annotation terms "DNA or RNA binding" and "transcriptional factor activity" are enriched as it is well-known that plant miRNAs are biased toward to target TFs and other regulatory genes [24]. Functional annotation of "response to abiotic or biotic stimulus" and "response to stress" are also significantly enriched (the corrected P-values < 1e-10), which is consistent with the fact that miRNAs play important roles in plant responses to environmental stresses as well as in development and genome maintenance [34,32].

**Table 2:** The functional enrichment for the 213 genes in GO annotation

GO annotation	Within group (213 genes)	All genes (25,676 genes)	P-Value
DNA or RNA binding	41	2801	8.8e-003
Transcription factor activity	62	3212	5.1e-009
Transcription	41	2466	5.0e-004
Nucleus	57	3087	1.9e-007
Transport	43	2780	1.8e-003
Response to abiotic or biotic stimulus	97	3911	5.1e-024
Response to stress	47	1821	9.9e-011

The P-values were adjusted for multiple tests using Bonferroni correction.

## Conclusion

Aiming at integrating transcription factor binding motifs and posttranscriptional regulatory motifs toward a better quantitative modeling of changes in mRNA level, we proposed a probabilistic approach to determine the context-dependent role of genomic TF binding motifs together with miRNA binding motifs in transcriptional and posttranscriptional regulation. Regardless the simple strategy employed, our method may provide an incomplete or coarse-grained portrait of the underlying transcriptional and posttranscriptional regulatory network. Consequently, our method facilitated the incorporation of diverse sources with limited prior knowledge. The relationship between sequence motifs and gene expression profiles could be investigated more precisely from datasets that observe expression profiles of miRNAs, mRNAs and proteins from the same samples simultaneously. Other posttranscriptional mechanisms, such as alternative splicing, may also be taken into considerations in the further network construction.

## Methods

### Dataset

The 212 *Arabidopsis *mature miRNA sequences were downloaded from miRBase (Release 12.0) released in September 2008 [35]. The 19 miRNA sequences in Release 3.0 were used to generate the training set of potential miRNA targets for the HMM of miRNA target prediction.

The entire intergenic region or 3000 bp, whichever was shorter, in the upstream of the TSS for each *Arabidopsis *gene was retrieved from TAIR (The Arabidopsis Information Resource) released in Mar 2006, and sequences of all the *Arabidopsis *transcripts were retrieved from the same site. GO annotation file of *Arabidopsis *genes was also downloaded from TAIR released in April 2007.

The *cop1 *mutant time course microarray dataset was kindly provided by Prof Deng Xingwang's lab in Yale Department of Biology. Both wildtype (reference sample) and *cop1 *mutant (test sample) were grown at 30 degree for a 10 time periods (0 hrs, 12 hrs, 24 hrs, 36 hrs, 48 hrs, 60 hrs, 72 hrs, 4 days, 5 days and 6 days) before transferred to 22 degree. The protocols for hybridization to the *Arabidopsis *microarray, microarray slide washing, and scanning were as described previously in Ma et al. [36]. Microarray spot intensity signals were acquired by using Axon GenePix Pro 3.0 software package (Axon Instruments Inc). The ratios were the expression intensities of *cop1 *mutant divided by that of wild type, respectively. The microarray time course gene expression data can be downloaded from . Average normalized log-transformed expression ratios of 5,689 genes were subjected to clustering analysis.

### Clustering and motif finding

To take into account the temporal relationship between time points, a HMM based approach, GQLCluster [37], was chosen for clustering analysis. The related software was downloaded from: . BIC (Bayesian Information Criterion) was used to determine the 'optimal' number of clusters for the dataset, and the 5,689 genes were divided into 12 clusters. AlignACE [38] was then used to detect overrepresented sequence motifs (TFBS candidates) in the 3000 bp upstream of the genes in the same cluster. The upstream sequences of all the genes were scanned using ScanACE for the motifs found by AlignACE [4].

### Potential miRNA targets prediction using HMM

**Figure 3:**
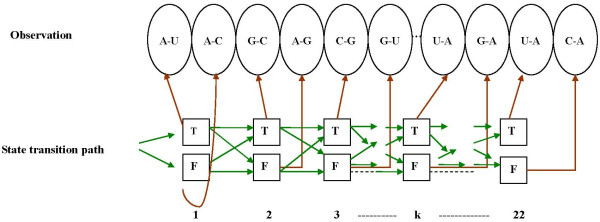
**An exemplar diagram of the inhomogeneous HMM**. Hidden states are defined over the binary space {*T*, *F*}, where *T *means a true matching state and a matching state could generate A-U, U-A, G-C or C-G as an emission symbol. *F *means a false matching state and a false matching state could emit one of the remaining combinations except the aforementioned four symbols. The position specific transition probabilities and emission probabilities would be estimated using a training-set of potential miRNA targets. (The transition probabilities and emission probabilities shown in the diagram were arbitrarily assigned.)

In our HMM model, hidden states are defined over the binary space {*T*, *F*}, where *T *means a true matching state, namely an endogenous miRNA needs to match to its target on the specific site. A matching state could generate A-U, U-A, G-C or C-G as an emission symbol. *F *means a false matching state, namely a miRNA dos not need to match to its target on this specific site. A false matching state could emit one of the remaining combinations except the aforementioned four symbols (Figure 3). Two types of probabilities need to be estimated: transition probabilities and emission probabilities. These probabilities are position specific in the inhomogeneous HMM. The parameters were estimated from a training set of the potential targets with up to 4 mismatches to one of the 19 miRNAs. Baum-Welch algorithm was used to update the parameters in the model until it reached (local) maximal log likelihood [39]. Convergence of the negative log-likelihood was checked up to a precision of 1e-12.

The Viterbi algorithm was used to find the most probable (optimal) state transition paths in the HMM [39]. We got 103 optimal paths in total after removing the redundant ones. The experimentally verified miRNAs and their optimal state paths obtained above were then used to scan for miRNA target motifs in the *Arabidopsis *genome.

The HMM was implemented as a Perl script and a genome-scale scanning for miRNA targets took about 10 hrs on a UNIX work station with 2 GHz processor and 2 G memory.

We used the cross-species conservation and minimum binding energy of miRNA/mRNA duplex as two additional filters to reduce the false positives in our HMM prediction. If a predicted *Arabidopsis *miRNA target can be mapped to a rice cDNA with the matched region longer than 15 bps and the identity higher than 80%, we keep this predicted target for further analysis. In the second round of filtering, we used RNAcofold [40] to calculate the minimum binding energy of miRNA/target duplex, and we only keep a predicted target when its minimum binding energy is less than -15 kcal/mol.

### Building Bayesian network

We followed the approach established by Beer and Tavazoie [4] and considered two layer networks with parent nodes representing sequence motifs (TFBS or miRNA target motifs) and descendent nodes representing gene expression patterns. Edges are directed and connected only from sequence elements to expression profiles. The network structure could be described with a 0–1 matrix, with *M *rows, as many as genes under consideration, and *N *columns, where *N *is the number of nodes [41].

The descendent nodes are gene expression pattern *v*_*c*_, where *c *= 1, 2,...., *C*, and *C *is the total number of clusters (expression patterns). The parent nodes are TFBSs with specific constraints or miRNA target motifs. The constraint of a TFBS is its orientation, its distance to TSS, and the presence or absence of other TFBSs. If two or more TFBSs are present, the interactive constraints are the distance between them, and/or their order relative to TSS, respectively. Let *ω *= (*σ*_1_, *σ*_2_,..., *σ*_*K*_) be the sequence constraints. If a constraint *n *is satisfied for a particular gene, then we have *σ*_*n *_= 1, otherwise *σ*_*n *_= 0. The final networks encode the distribution of *P*(*v*_*c*_|*σ*_1_, *σ*_2_,..., *σ*_*K*_), namely the probability of the gene being a member (*v*_*c *_= 1) or not being a member (*v*_*c *_= 0) of the cluster *c*, given the states of the sequence constraints *ω*. About 80% of the total genes were used as training set and the rest 20% genes were used as testing set [4].

From Bayes' theorem, we have:



where *D *is the data and *S *is the network structure. In our case, a network was learnt for each cluster. Assuming unrestricted multinomial distribution, parameter independence, Dirichlet priors and complete data, the *p*(*D*|*S*) was given by



where *r *is the number of unique instantiations for each descent node, so we have *r *= 2, and *q *is the number of parent instantiations. We use *N*_*jk *_to denote the number of cases in *D *in which variable *v*_*c *_has the value *k *and its parent was instantiated as *j*, and . We assume uniform priors, such that *a*_*jk *_= 1 and . Parents are added progressively to a node until no additional parent could increase the structure probability [42].

A model with the highest log marginal likelihood (or the highest posterior probability, assuming equal priors on structure) is the best sequential predictor of the data *D*. For any given gene, the probability that this gene is a member of cluster *c *could be calculated by [43]:



where j* is the parent instantiate of the network structure for gene expression pattern *c *and *k *= 1.

The algorithms for Bayesian network building and gene expression pattern prediction were implemented as C++ programs and the total runtime is about 1 hour on a desktop PC with 1 G memory.

### Enrichment of functional annotation terms from Gene Ontology

Genes with the same annotation terms from Gene Ontology (GO) were grouped. The size of each group was compared to the total number of genes having the same GO annotation term in the *Arabidopsis *genome. P-value, which indicated the significance of enrichment, is calculated from the hypergeometric tail [44,45]:



where *C *is the number of genes with a particular GO annotation term in the *Arabidopsis *genome, *G *is the total number of genes in *Arabidopsis *which is 25,676, *c *is the number of genes in a group with the particular GO annotation term and *g *is the total number of genes in that group. In our case, *g *is 213. The P-value was adjusted for multiple tests using Bonferroni correction.

### Availability and requirements

The C++ and Perl programs that implement the methods in this work are available for download from our supplemental data web page , and a README file can be found in the package for the instructions to run these programs. Additional files are available in the above web site: Tables S1 and S2 listed TFBSs and miRNA target nodes adopted in the networks, respectively. Table S3 listed the known motifs that were adopted by the networks. The COP1 microarray time course gene expression data, the 12 gene clusters and their mean expression profiles, the simulation results of miRNA target prediction as well as the predicted TFBSs for each of the 12 gene cluster are also available for download.

## Authors' contributions

LL developed the method, implemented the algorithms, carried out the data analysis, and drafted the manuscript. JL conducted the supervision of the study, involved in the methodology development and manuscript preparation. All authors read and approved the final manuscript.

## References

[B1] Hvidsten TR, Wilczynski B, Kryshtafovych A, Tiuryn J, Komorowski J, Fidelis K (2005). Discovering regulatory binding-site modules using rule-based learning. Genome Res.

[B2] Elemento O, Tavazoie S (2005). Fast and systematic genome-wide discovery of conserved regulatory elements using a non-alignment based approach. Genome Biology.

[B3] Segal E, Friedman N, Kaminski N, Regev A, Koller D (2005). From signatures to models: understanding cancer using microarrays. Nature Genetics.

[B4] Beer MA, Tavazoie S (2004). Predicting Gene Expression from Sequence. Cell.

[B5] Bar-Joseph Z, Gerber GK, Lee TI, Rinaldi NJ, Yoo JY, Robert F, Gordon DB, Fraenkel E, Jaakkola TS, Young RA (2003). Computational discovery of gene modules and regulatory networks. Nat Biotechnol.

[B6] Levine E, Zhang Z, Kuhlman T, Hwa T (2007). Quantitative Characteristics of Gene Regulation by Small RNA. PLoS Biology.

[B7] Hobert O (2008). Gene regulation by transcription factors and microRNAs. Science.

[B8] Bartel DP (2004). MicroRNAs: Genomics, Biogenesis, Mechanism, and Function. Cell.

[B9] Chan CS, Elemento O, Tavazoie S (2005). Revealing Posttranscriptional Regulatory Elements Through Network-Level Conservation. PLoS Computational Biology.

[B10] Arnim AGv, Deng X-W (1994). Light inactivation of arabidopsis photomorphogenic repressor COP1 involves a cell-specific regulation of its nucleocytoplasmic partitioning. Cell.

[B11] Neff MM, Fankhauser C, Chory J (2000). Light: an indicator of time and place. Genes Dev.

[B12] Shin B, Choi G, Yi H, Yang S, Cho I, Kim J, Lee S, Paek N-C, Kim J-H, Song P-S (2002). AtMYB21, a gene encoding a flower-specific transcription factor, is regulated by COP1. The Plant Journal.

[B13] Osterlund MT, Ang L-H, Deng XW (1999). The role of COP1 in repression of Arabidopsis photomorphogenic development. Trends in Cell Biology.

[B14] Shin B, Choi G, Yi H, Yang S, Cho I, Kim J, Lee S, Paek N-C, Kim J-H, Song P-S (2002). AtMYB21, a gene encoding a flower-specific transcription factor, is regulated by COP1. The Plant Journal.

[B15] Miséra S, Müller AJ, Weiland-Heidecker U, Jürgens G (1994). The FUSCA genes of Arabidopsis: negative regulators of light responses. Mol Gen Genet.

[B16] Kwok SF, Piekos B, Misera S, Deng XW (1996). A Complement of Ten Essential and Pleiotropic Arabidopsis COP/DET/FUS Genes Is Necessary for Repression of Photomorphogenesis in Darkness. Plant Physiol.

[B17] Wei N, Deng XW (1996). The Role of the COP/DET/FUS Genes in Light Control of Arabidopsis Seedling Development. Plant Physiol.

[B18] Ma L, Gao Y, Qu L, Chen Z, Li J, Zhao H, Deng XW (2002). Genomic Evidence for COP1 as a Repressor of Light-Regulated Gene Expression and Development in Arabidopsis. Plant Cell.

[B19] Roth FP, Hughes JD, Estep PW, Church GM (1998). Finding DNA regulatory motifs within unaligned noncoding sequences clustered by whole-genome mRNA quantitation. Nature Biotechnology.

[B20] Jones-Rhoades MW, Bartel DP (2004). Computational Identification of Plant MicroRNAs and Their Targets, Including a Stress-Induced miRNA. Molecular Cell.

[B21] Schwab R, Palatnik JF, Riester M, Schommer C, Schmid M, Weigel D (2005). Specific Effects of MicroRNAs on the Plant Transcriptome. Developmental Cell.

[B22] Bartel B, Bartel DP (2003). MicroRNAs: At the Root of Plant Development?. Plant Physiol.

[B23] Miranda KC, Huynh T, Tay Y, Ang Y-S, Tam W-L, Thomson AM, Lim B, Rigoutsos I (2006). A Pattern-Based Method for the Identification of MicroRNA Binding Sites and Their Corresponding Heteroduplexes. Cell.

[B24] Jones-Rhoades MW, Bartel DP, Bartel B (2006). MicroRNAs and their regulatory roles in plants. Annual Review of Plant Biology.

[B25] Schliep A, Steinhoff C, Schonhuth A (2004). Robust inference of groups in gene expression time-courses using mixtures of HMMs. Bioinformatics.

[B26] Gao Y, Li J, Strickland E, Hua S, Zhao H, Chen Z, Qu L, Deng XW (2004). An Arabidopsis Promoter Microarray and its Initial Usage in the Identification of HY5 Binding Targets in Vitro. Plant Molecular Biology.

[B27] Lee J, He K, Stolc V, Lee H, Figueroa P, Gao Y, Tongprasit W, Zhao H, Lee I, Deng XW (2007). Analysis of Transcription Factor HY5 Genomic Binding Sites Revealed Its Hierarchical Role in Light Regulation of Development. Plant Cell.

[B28] Bussemaker HJ, Li H, Siggia ED (2001). Regulatory element detection using correlation with expression. Nature Genetics.

[B29] Li Y, Lee KK, Walsh S, Smith C, Hadingham S, Sorefan K, Cawley G, Bevan MW (2006). Establishing glucose- and ABA-regulated transcription networks in Arabidopsis by microarray analysis and promoter classification using a Relevance Vector Machine. Genome Res.

[B30] Foat BC, Houshmandi SS, Olivas WM, Bussemaker HJ (2005). Profiling condition-specific, genome-wide regulation of mRNA stability in yeast. PNAS.

[B31] Rajewsky N (2006). microRNA target predictions in animals. Nature Genetics.

[B32] Sood P, Krek A, Zavolan M, Macino G, Rajewsky N (2006). Cell-type-specific signatures of microRNAs on target mRNA expression. PNAS.

[B33] Rhoades MW, Reinhart BJ, Lim LP, Burge CB, Bartel B, Bartel DP (2002). Prediction of plant microRNA targets. Cell.

[B34] Sunkar R, Zhu J-K (2004). Novel and Stress-Regulated MicroRNAs and Other Small RNAs from Arabidopsis. Plant Cell.

[B35] Griffiths-Jones S, Grocock RJ, van Dongen S, Bateman A, Enright AJ (2006). miRBase: microRNA sequences, targets and gene nomenclature. Nucl Acids Res.

[B36] Ma L, Li J, Qu L, Hager J, Chen Z, Zhao H, Deng XW (2001). Light Control of Arabidopsis Development Entails Coordinated Regulation of Genome Expression and Cellular Pathways. Plant Cell.

[B37] Schliep A, Schonhuth A, Steinhoff C (2003). Using hidden Markov models to analyze gene expression time course data. Bioinformatics.

[B38] Hughes JD, Estep PW, Tavazoie S, Church GM (2000). Computational identification of Cis-regulatory elements associated with groups of functionally related genes in Saccharomyces cerevisiae. Journal of Molecular Biology.

[B39] Durbin R, Eddy S, Krogh A, Mitchison G (1998). Biological sequence analysis – Probabilistic models of proteins and nucleic acids.

[B40] Zuker M (2003). Mfold web server for nucleic acid folding and hybridization prediction. Nucl Acids Res.

[B41] Sabatti C, James GM (2006). Bayesian sparse hidden components analysis for transcription regulation networks. Bioinformatics.

[B42] Cooper GF, Herskovits E (1992). A Bayesian Method for theInduction of Probabilistic Networks from Data. Machine Learning.

[B43] Heckerman D (1995). A Tutorial on Learning with Bayesian Networks.

[B44] Tavazoie S, Hughes JD, Campbell MJ, Cho RJ, Church GM (1999). Systematic determination of genetic network architecture. Nat Genet.

[B45] Das D, Nahle Z, Zhang MQ (2006). Adaptively inferring human transcriptional subnetworks. Mol Syst Biol.

